# Performance nutrition for cold-weather military operations

**DOI:** 10.1080/22423982.2023.2192392

**Published:** 2023-03-19

**Authors:** Lee M. Margolis, Stefan M. Pasiakos

**Affiliations:** a Military Nutrition Division, U.S. Army Research Institute of Environmental Medicine, Natick, MA, USA; b Military Performance Division, U.S. Army Research Institute of Environmental Medicine, Natick, MA, USA

**Keywords:** Energy balance, energy expenditure, protein balance, physical performance, military

## Abstract

.High daily energy expenditure without compensatory increases in energy intake results in severe energy deficits during cold-weather military operations. The severity of energy deficits has been proportionally linked to declines in body mass, negative protein balance, suppression of androgen hormones, increases in systemic inflammation and degraded physical performance. Food availability does not appear to be the predominant factor causing energy deficits; providing additional rations or supplement snack bars does not reduce the severity of the energy deficits. Nutrition interventions that allow greater energy intake could be effective for reducing energy deficits during cold-weather military operations. One potential intervention is to increase energy density (i.e. energy per unit mass of food) by increasing dietary fat. Our laboratory recently reported that self-selected higher energy intakes and reductions in energy deficits were primarily driven by fat intake (*r* = 0.891, *r^2^* = 0.475), which, of the three macronutrients. Further, soldiers who ate more fat lost less body mass, had lower inflammation, and maintained net protein balance compared to those who ate less fat. These data suggest that consuming high-fat energy-dense foods may be a viable nutritional intervention that mitigates the negative physiological effects of energy deficit and sustains physical performance during cold-weather military operations.

## Introduction

Sustained periods of high daily energy expenditure coupled with inadequate energy intake are common during most military training and combat operations. Failure to adequately compensate for the high energy expenditure with greater energy intake occurs because there may be inadequate food availability, as the amount of food a service member has to consume may be limited to what they can carry, limited time to eat, the palatability of the foods provided is low and that appetite is suppressed [1]. Imbalance between energy intake and expenditure, known as negative energy balance (energy expenditure>energy intake) [2–5], results in undesired reductions in body and muscle mass [5–8], altered substrate oxidation, negative protein balance (protein synthesis<protein breakdown), suppressed androgen hormones and increased systemic inflammation, which collectively occur with concomitant decrements in physical performance [2–5,8–12].

Exposure to environmental extremes can exacerbate the severity of energy deficits (i.e. percentage of energy intake needed to establish energy balance) and result in physiological consequences that may ultimately lead to greater decrements in physical performance during military operations [13]. Cold-weather operations result in extremely high energy expenditure, ranging from 5000 to 7000 kcal/d, due to sustained physical activity and increased energy cost of locomotion while traversing snowy and mountainous terrain [3,4,14,15]. Cold-weather operations further complicate the ability to eat enough food and achieve energy balance, as food needs to be thawed and often reconstituted with water produced by melting snow. The combination of high energy expenditures and barriers to eating in cold-weather environments results in suboptimal energy intakes, resulting in energy deficits as high as 70% of total energy needs [3,4].

Given the potential for increased operations in arctic regions of the world, there is an urgent need to develop viable nutritional interventions to sustain performance during cold-weather operations by limiting the severity of energy deficits incurred and their associated consequences. This manuscript will provide a brief overview of contemporary research from our laboratory assessing the metabolic and physiological consequences of cold-weather operations. Additionally, this manuscript will outline how results from our previous studies may be used to inform the development of the next generation of cold-weather rations and field-fielding strategies that improve voluntary energy intake during arduous cold-weather operations and thereby minimise the severity of energy deficits and consequent physical performance decline.

## Metabolic responses to cold

**Figure 1.**
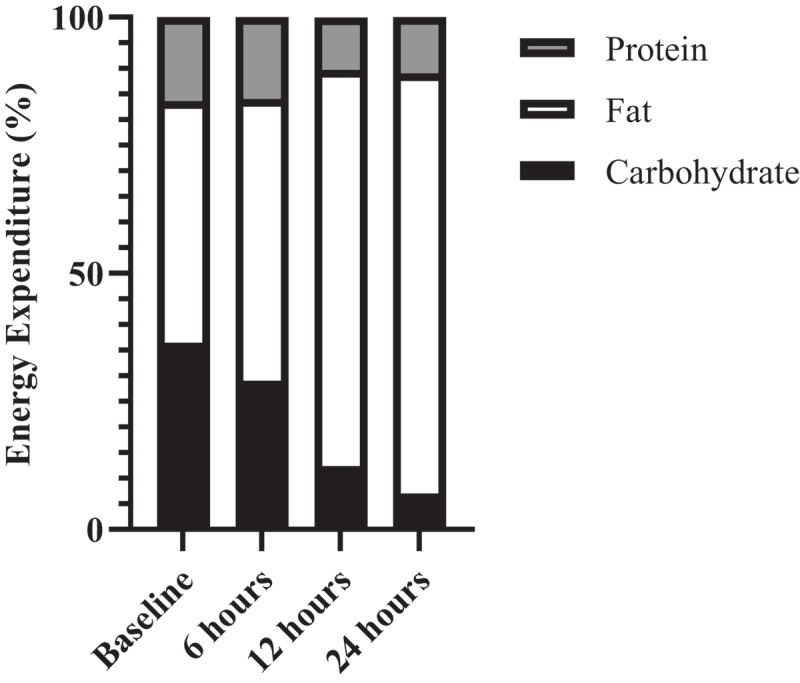
Adapted from Haman et al., 2016 [20]. Temporal metabolic adaptations to 24 hours of cold-weather exposure.

Metabolic adaptations to cold-weather exposure are variable and depend on multiple factors, such as ambient temperature, wind speed, water exposure, clothing, anthropometrics, sex, race, energy status and fitness level [13]. In general, shivering thermogenesis prevents decreases in core body temperature by increasing energy expenditures at rest in unacclimatised individuals exposed to cold-weather [16]. These involuntary muscle contractions are fuelled by increases in both carbohydrates and fat oxidation during acute (<4 hours) cold-weather exposure [17,18]. During this initial phase of cold-weather exposure, both plasma glucose and muscle glycogen oxidation rates nearly double, and muscle glycogen accounts for 75% of the oxidised carbohydrate [17,19]. Concurrently, fat oxidation is fourfold greater while shivering and accounts for a greater proportion of total body heat production compared to that provided by carbohydrate [17]. As cold-weather exposure is sustained, fat oxidation continues to increase over the initial 24-hour exposure period (Figure 1) [20]. Conversely, sustained cold-weather exposure leads to reductions in both protein and carbohydrate oxidation, likely to spare muscle mass (i.e. the body’s only repository of essential amino acids) and glycogen stores.

Thermogenesis during cold-weather exposure may also be initiated voluntarily through physical activity. Similar to resting conditions, discrepancies in metabolic rate appear when individuals conduct low-intensity aerobic exercise (40% VO_2_max) under cold or temperate ambient conditions [21]. Muscle glycogen stores decline to a greater extent when performing low-intensity aerobic exercise during acute cold-weather exposure than they do during the same exercise in temperate conditions [21]. Differences in glycogen fuel use may result from inadequate heat production, resulting in shivering thermogenesis to persist during low-intensity aerobic exercise with acute cold-weather exposure. Interestingly, during moderate-to-high (>60% VO_2_max) aerobic exercise, carbohydrate oxidation is maintained or decreased, while fat oxidation is increased under cold or ambient temperatures [21–26]. While the specific mechanism is not fully understood, increased fat oxidation during aerobic exercise in cold-weather conditions has been suggested to result from increased intramuscular triglyceride oxidation and transport of long-chain fatty acids across the mitochondrial members [26]. It should be noted that all these exercise interventions were conducted under acute cold-weather conditions in a controlled laboratory setting aimed at inducing cold strain. Metabolic and physiological adaptations to cold may differ under “real-world” environmental exposure because wearing appropriate clothing and being continuously physically active likely maintain core and skin temperatures and negate any potential cold strain and changes in substrate oxidation [27]. Beyond clothing and physical activity, other factors such as the amount and timing of energy and macronutrient intake and gastrointestinal effects during “real-world” cold-weather operations [28] may result in metabolic responses that differ from controlled laboratory results.

## Energy deficit and physiological decrements

**Figure 2.**
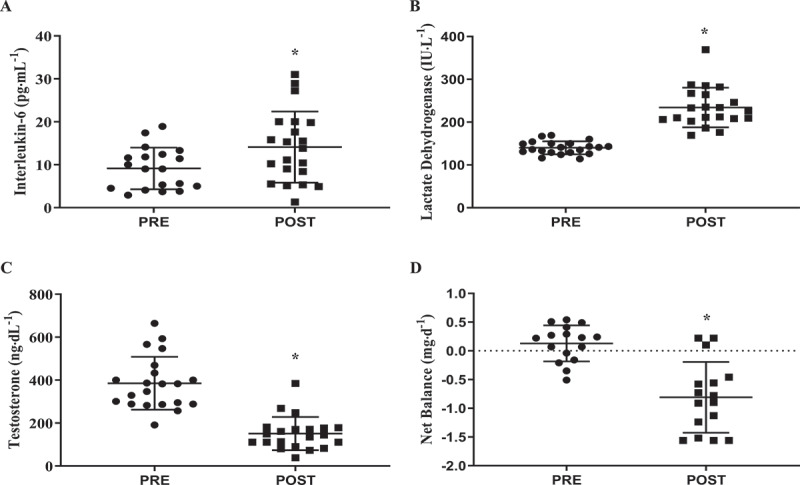
Adapted from Margolis et al., 2014 [4]. Physiological decrements following a 7-day cold-weather operation. *significantly lower POST compared to PRE; p<0.05.

In a recent observational longitudinal study conducted in young healthy Norwegian conscript soldiers, we reported [4,10] that following a 7-day cold-weather training exercise (ambient temperature range: −26°C to −6°C), participants lost 3% of their body mass resulting from 55% average energy deficits (2853 kcal/d). Energy deficits were determined by assessing daily energy expenditures using the doubly-labelled water technique, while energy intake was measured from a collection of daily ration trash and food logs. The deficits caused increased inflammation (Figure 2A) and muscle damage (Figure 2B) and reduced androgen hormone concentrations (Figure 2C) and whole-body net protein balance (Figure 2D) at the end of the 7-day cold-weather training exercise compared to baseline values. The severity of the energy deficit was the result of high daily energy expenditures, ranging from 5,480 kcal/d during 4 days of military-specific training (e.g. mountainous terrain navigation and general winter survival training) to 6,851 kcal/d during 3 days of a 54-km ski march where soldiers carried an additional 45 kg of equipment. Interestingly, during the first 4 days of training, soldiers were provided with three Norwegian arctic combat rations (3,842 kcal/d) and four arctic combat rations (5,177 kcal/d) during the last 3 days. Although an additional 1335 kcal/d was provided during the second half of the training, soldiers energy intake only increased by 363 kcal/d during the ski march compared to the military-specific training portion of the training exercise [4]. The soldiers chose not to consume 35% of the total energy provided to them, despite the increase in daily energy expenditures during the 54-km ski march. These data suggest that food availability was not a predominant factor contributing to the energy deficit during the cold-weather training exercise.

**Figure 3.**
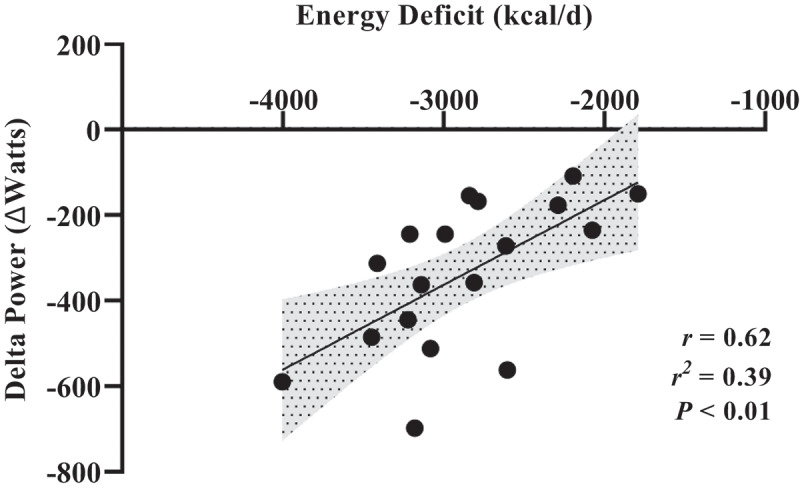
Adapted from Margolis et al 2014 [4]. Association between energy balance and delta (PRE minus POST) lower-body power during 7-day cold-weather operation.

Severe energy deficits during cold-weather military training operations not only disrupted physiological homoeostasis but were also associated with a 7% (330 W) decline in lower-body peak power, as determined by a countermovement vertical jump test conducted before and after the training operation [4]. Those who experienced higher total daily energy deficits also experienced greater declines in lower-body power (r^2^=0.39; Figure 3) than those with lower deficits. As lower-body power is a strong predictor of military-specific tasks [29–32], exacerbated declines due to greater energy deficit may compromise mission success. The proportional link between performance and energy deficit suggests that minimising energy deficits and not necessarily achieving energy balance, by increasing energy intake may be critical for maintaining performance. A predictive equation using linear regression suggests that if soldiers achieved an energy deficit of −1166 kcal/d (19% total energy needs), then performance is maintained. These data suggest that there is an acceptable degree of energy deficit during cold-weather operations that will not compromise physical performance. However, it should be noted that energy deficit only accounted for 39% of the variance in changes in lower-body power, indicating that other factors such as fatigue, muscle soreness and motivation may contribute to the other 61% of the variance that can be not explained by energy deficit.

In a follow-up study under similar environmental conditions (ambient temperature range: −17°C to −3°C), our group attempted to reduce energy deficits and minimise physiological decrements by supplementing rations with snack bars. Norwegian soldiers were provided with three arctic combat rations (3,487 kcal/d), three arctic combat rations plus carbohydrate snack bars (4,545 kcal/d) or three arctic combat rations plus protein snack bars (4,549 kcal/d) during a 4-day cold-weather training exercise, where soldiers skied 51 km carrying an additional 45 kg of equipment [3]. Eat-on-the-move snack bars were chosen because Norwegian soldiers in our first study consumed more than 90% of the bars provided in their combat rations [4]. Similar to our first study, the 4-day cold-weather operation induced high daily energy expenditures, with no difference between carbohydrate (6,181 kcal/d), protein (6,167 kcal/d) and control (6,096 kcal/d) groups [3]. Although participants in the protein and carbohydrate snack bar groups consumed 82% and 85%, respectively, of the bars provided to them, there was no difference in energy deficits between carbohydrate (−3,050 kcal/d), protein (−3,402 kcal/d) and control (−3,595 kcal/d) groups [3]. The lack of a dietary treatment effect was due to soldiers in snack bar groups choosing to consume the bars over items provided in Norwegian Arctic combat rations. Similar to when soldiers were provided with an extra combat ration per day in our initial study [4], soldiers in the snack bar groups failed to consume ~ 35% of the total energy that was provided to them [3]. Independent of dietary treatment, soldiers were in negative protein balance, increased fat oxidation and ketogenesis and demonstrated increased inflammation (e.g. interleukin-6 and hepcidin) at the conclusion of the 4-day cold-weather training exercise [3,9,33]. Ineffectiveness of supplement protein foods to decrease the severity of energy deficit and subsequent physiological decrements has been corroborated by other laboratories assessing the impact of supplement protein intake during 8 and 10-day Norwegian cold-weather operations [34,35].

**Figure 4.**
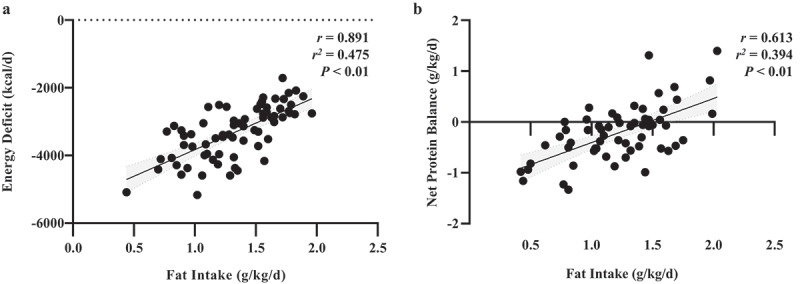
Adapted from Margolis et al., 2016 [3]. Association between energy deficit (**a**) and net protein balance (**b**) to relative fat intake during 4-day cold-weather operation.

Despite the null results from the snack bar interventions in our second study [3], there was substantial inter-individual variability in the amount of energy soldiers consumed. Assessment of food choices in our previous work revealed top reasons for soldiers not consuming certain food items were insufficient time to eat, not liking food/beverage and not being hungry (unpublished data). The lack of hunger ques when energy deficit is induced by increases in energy expenditure agrees with controlled laboratory studies [36,37]. Similarly, Ahmed et al. [38] reported that energy intake of Canadian Armed Forces did not differ following exercise under cold-weather conditions (−10°C) compared to being sedentary under temperate conditions (−21°C). This absence of increased hunger ques resulting in no difference in energy intake is an important contributor to energy deficits during field operations. Importantly, soldiers who self-selected higher energy intakes were less inflamed, lost the least weight and retained more protein than those who ate less [3,9], providing further evidence that minimising energy deficits during cold-weather operations is fundamentally the most important aspect of cold-weather feeding strategies. Interestingly, while intake of all three macronutrients were associated with energy deficit, fat intake accounted for 48% (Figure 4a) of the variance in energy deficit, while carbohydrate accounted for 44% (r=0.875, r^2^=0.436) and protein for only for 8% (r=0.528, r^2^=0.079). In addition, fat intake accounted for 39% (Figure 4b) of the variance in net protein balance and was more strongly associated with protein retention than carbohydrate (r=0.427, r^2^=0.182) and protein (r=0.443, r^2^=0.196).

## Increasing energy density to attenuate energy deficit

Given that fat intake was the macronutrient that was most highly associated with energy deficit and net protein balance in our second study, increasing the fat content of rations may be a viable intervention to reduce energy deficits and negative physiological consequences during cold-weather operations. Fat may be a more advantageous macronutrient during periods of high-energy expenditure simply because it provides 9 kcal/g, while carbohydrate and protein only provide 4 kcal/g. The greater energy density, defined as the energy content per unit mass of a food [39], allows for more energy to be consumed in a small volume of food. The top reasons reported by soldiers not consuming ration items in our second study were insufficient time to eat, not liking food/beverage and not being hungry (unpublished data). The latter effect is interesting since the energy deficit stimulates biological responses that are responsible for increasing appetite to prevent further weight loss and promote weight regain [40,41]. However, when energy deficits are caused largely by physical activity-induced energy expenditure, hunger does not always increase [42–44] and controlled laboratory studies have shown that this can blunt energy intake, resulting in increased energy deficits [36,37]. Energy-dense high-fat foods may overcome several factors that limited food intake during cold-weather operations. High-fat foods are generally rated as more palatable, promote energy intake [45], are less filling than foods high in protein and carbohydrate [46] and may be less likely to exacerbate exercise-induced appetite suppression. Thus, generation of a more palatable high-fat energy-dense ration product may overcome the lack of increased hunger ques observed during strenuous cold-weather military operations to improve energy intake and minimise energy deficits.

Manipulating the energy density of foods without changing food volume is an extremely effective approach for altering short-term energy intake across a variety of populations [39,47–49]. There is evidence to support a proportional increase in energy intake when the energy density of diet increases. Specifically, increasing the energy density of a diet by 34% has been shown to result in a 29% increase in daily energy intake and ~1 kg weight gain over 2 weeks in healthy adults [50]. Further, increasing the energy density of a single meal by 36% results in a 33% increase energy intake without any effect on hunger ques or the hunger-stimulating hormone ghrelin [51]. Increasing the energy density of cold-weather military rations should result in proportional increases in energy intake, which should reduce energy deficits and sustain physical performance in the cold. Greater energy density may be achieved by increasing the fat content of entrées and side items already present in cold-weather rations. Additionally, there are viable ready-to-use products that are either commercially available or already provided by the US Army Cold-Weather Rations (Table 1).

**Table 1.** Examples of energy-dense food products.

Mixed Nuts	Freeze Dried Cheese	Cheese Spreads	Dessert Bars
Chocolate	Cheese	Peanut Butter	Beef Snacks
Salami	Pemmican	Plumpy’Nut®	Truffles

Examples of energy dense food products.

There is historical precedent within the military to naturally shift macronutrient intakes towards higher fat consumption while operating in cold-weather environments. Jones and Lee as part of the Institute of Medicine, Committee on Military Nutrition Research, highlighted that without a deliberate intervention to alter macronutrient intake patterns, service members stationed in cold-weather environments tended to self-select dietary fat at 35–40% of total daily energy consumed [52]. With increased fat intake, carbohydrate accounted for 45–50% of total daily energy, while protein constituted ~15% [52]. This modest increase in dietary fat intake may be sufficient to increase energy intake to minimise or negate energy deficit-induced declines in physical performance [1,3]. Furthermore, as carbohydrate remains the predominate macronutrient consumed daily, elevations in fat intake are unlikely to result in significant declines in muscle glycogen stores that would negatively impact physical performance [53].

## Conclusion

In conclusion, cold-weather military operations result in high daily energy expenditures due to sustained physical activity. Soldiers typically fail to adequately increase energy intake and achieve energy balance. This results in energy deficits during cold-weather military operations that lead to negative physiological effects that ultimately reduce physical performance. Work from our laboratory has identified the possibility that higher-fat energy-dense food products may minimise the severity of energy deficits and sustain physical performance. To improve energy balance, minimal increases in dietary fat intake, accounting for 35–40% of the total energy consumed daily, may increase energy intake. However, which ration products should be modified to increase their energy density has not been studied nor has the influence of elevated fat intake on physical performance during cold-weather operations. Future investigation is warranted to understand the impact of consuming high-fat energy-dense ration products under both controlled laboratory and “real-world” cold-weather military operations on substrate oxidation and physical performance.
